# Functional leg performance 2 years after ACL surgery: a comparison between InternalBrace™-augmented repair versus reconstruction versus healthy controls

**DOI:** 10.1186/s10195-023-00723-5

**Published:** 2023-09-21

**Authors:** Linda Bühl, Sebastian Müller, Corina Nüesch, Geert Pagenstert, Annegret Mündermann, Christian Egloff

**Affiliations:** 1 Department of Orthopaedics and Traumatology, University Hospital Basel, Spitalstrasse 21, 4031 Basel, Switzerland; 2 Department of Biomedical Engineering, University of Basel, Basel, Switzerland; 3 Department of Clinical Research, University of Basel, Basel, Switzerland; 4 Department of Spine Surgery, University Hospital Basel, Basel, Switzerland; 5 Clarahof Clinic of Orthopaedic Surgery, Basel, Switzerland

**Keywords:** ACL repair, InternalBrace, ACL reconstruction, Postural stability, Knee performance, ACL rehabilitation, Proprioception

## Abstract

**Background:**

While clinical and patient-reported outcomes have been investigated in patients after InternalBrace™-augmented anterior cruciate ligament repair (ACL-IB), less is known regarding restoration of functional performance. We aimed to determine differences in functional performance within and between patients 2 years after ACL-IB, patients 2 years after ACL reconstruction (ACL-R), and healthy controls.

**Materials and Methods:**

A total of 29 ACL-IB, 27 ACL-R (hamstring autograft), and 29 controls performed single-leg hop (maximum forward distance hop, SLH; side hop > 40 cm in 30 s, SH), proprioception (knee joint position sense at 30° and 60° flexion), and dynamic postural balance (Y Balance) tests. Differences were calculated within groups (side-to-side difference) and between the involved leg of patients and the non-dominant leg of controls, and were evaluated to predefined statistical (*P* < 0.05), clinically relevant, and methodological (smallest detectable change) thresholds. The number of exceeded thresholds represented no (0), small (1), moderate (2), or strong (3) differences. In addition, the relative number of participants achieving leg symmetry (≥ 90%) and normal performance (≥ 90% of the average performance of the non-dominant leg of controls) were compared between groups (chi-squared tests, *P* < 0.05).

**Results:**

We observed no-to-moderate leg differences within ACL-IB (moderate difference in hops) and within ACL-R (moderate difference in knee proprioception), no leg differences between patient groups, no-to-small leg differences between ACL-IB and controls, and no leg differences between ACL-R and controls in functional performance. However, two patients in ACL-IB and ACL-R, respectively, passed the hop pretest only with their uninvolved leg, and fewer patients after ACL-IB and ACL-R than controls reached a leg symmetry and normal leg performance of controls in SLH (*P* < 0.001).

**Conclusions:**

Functional performance seems to be comparable 2 years postoperatively between ACL-IB and ACL-R for a specific subgroup of patients (i.e., proximal ACL tears, moderate activity level). However, the presumed advantage of comparable functional outcome with preserved knee structures after augmented ACL repair compared with ACL-R, and the tendency of both patient groups toward leg asymmetry and compromised single-leg hop performance in the involved legs, warrants further investigation.

*Level of Evidence* Level III, case-control study.

*Trial registration* clinicaltrials.gov, NCT04429165 (12/09/2020). Prospectively registered, https://clinicaltrials.gov/ct2/show/NCT04429165.

## Introduction

The advantages of anterior cruciate ligament (ACL) repair compared with gold standard ACL reconstruction (ACL-R) are a less invasive operation, no tendon harvest, and preservation of the native ligament [50]. To ensure ligament healing and further improve outcomes, in recent years ACL repair with additional static tape augmentation (synthetical band) for proximal ACL tears has been introduced (ACL-IB; InternalBrace™, Arthrex Inc., Naples, Florida, USA) [54].

While the clinical, patient-reported and biomechanical-functional outcomes after ACL-R have been summarized in an umbrella review [2], to date, mainly clinical outcomes and patient-reported knee function (questionnaire-based) after augmented ACL repair have been reported [17, 53, 54] and compared with gold standard ACL-R [4, 7, 16, 18, 20, 24, 25, 28, 32, 43, 48, 51, 52]. Results include comparable return-to-sports rate [7, 32], sense of wellbeing [32], anxiety during sports [32], radiological [28], patient-reported outcome [7, 25, 28, 32, 48], less daily awareness of the operated knee [7, 52], an earlier return to full range of motion [51], higher rerupture rates [7], or comparable rerupture rates when including only patients aged > 21 years [7], as well as comparable [7, 20], lower [48], or higher anteroposterior laxity [25, 28] after ACL-IB compared with ACL-R.

Much less is known about the preservation or restoration of dynamic functional performance after augmented ACL repair. Recent studies have reported comparable [25] or better [7] hamstring strength results and comparable hop test performance [25] after ACL-IB compared with ACL-R. Preserving the native ACL presumably preserves blood supply and proprioception [50], entailing joint position sense (JPS), and contributes to normal joint function, including stiffness and stability [41]. To date, data on proprioception or dynamic postural control in patients after ACL-IB are lacking. Our aim was to determine differences in functional leg performance in hops, knee proprioception, and dynamic postural control (I) between the legs of patients 2 years after ACL-IB, the legs of patients 2 years after ACL-R, and the legs of healthy controls, and (II) between the involved leg of patients and the non-dominant leg of controls using statistical, clinical, and methodological thresholds. In addition, we aimed (III) to compare the number of participants who had normal leg symmetry and normal leg performance between groups.

## Methods

### Study design

This study is a substudy of a larger nonrandomized retrospective comparative study with prospective data collection [29] approved by the regional ethics board (Ethikkomission Nordwestschweiz EKNZ 2019-00491 and EKNZ 2020-00551) and registered at clinicaltrials.gov (NCT04429165).

### Participants

Hospital lists of ACL repair surgeries performed between May 2016 and March 2020 and lists of ACL reconstruction surgeries performed between March 2019 and April 2020 were screened according to our inclusion and exclusion criteria (Table 1) for eligible patients 2 years after unilateral primary ACL-IB after proximal ACL rupture (Sherman Classification Types I and II [45]), and eligible patients 2 years after unilateral primary single-bundle ACL-R using hamstring tendon autograft. For ACL-R, a maximum of 8 months between index injury and surgery was accepted to reduce the influence of manifested deficits in functional performance due to prior time or years of conservative therapy. Eligible healthy subjects of corresponding sex and age were recruited via flyers in the surrounding communities and announcements on online platforms. For each ACL-IB patient, one sex-matched patient after ACL-R and one sex-matched healthy subject with the smallest possible age difference were selected. The maximum difference in age between all groups was limited to no more than 4 years. On the basis of our sample size estimation [29], 28 subjects were required to detect a statistically significant difference with a power of 80% and a significance level of 5%.

**Table 1:** Inclusion and exclusion criteria for patients after ACL-IB, patients after ACL-R, and controls (Modified from the umbrella study protocol [29])

	*ACL-IB*	*ACL-R*	*Controls*
*Inclusion criteria*	2 years since surgery		No previous injury of lower extremity, menisci, or ligament apparatus of the knee
	*ACL repair and InternalBrace™-augmentation* for proximal ACL ruptures (Sherman Classification Types I and II [45]) operated within 5 weeks after index injury	*ACL reconstruction* with autologous hamstring (semitendinosus, and if needed gracilis) tendon operated the latest 8 months after index injury	
*Exclusion criteria*	Concomitant rupture of posterior cruciate ligament or complete rupture of both collateral ligaments Previous injury or surgical treatment of the injured leg within the past 6 months Previous surgical treatment of the contralateral leg		

*ACL-IB* anterior cruciate ligament repair and InternalBrace™ augmentation after proximal ruptures, *ACL-R* anterior cruciate ligament reconstruction

### Study procedures

Participants (patients and controls) provided informed consent, and completed the Tegner activity score (TAS) [49] and the subjective International Knee Documentation Committee (IKDC) [19] questionnaires, which served as a descriptive group parameter. Anthropometric and clinical parameters were recorded. Control subjects were asked to indicate their dominant leg, if possible, otherwise they were asked about their preferred kicking leg. Participants completed a 5-min warm-up walk on a treadmill at self-selected walking speed. They then completed knee JPS (proprioception performance), Y Balance (YB, dynamic postural control performance), single-leg hop (SLH), and side hop (SH, both hop performance) tests with both legs in their own shoes. The starting leg was randomized for each task, and legs were alternated for the trials of YB, SLH, and SH. Data were managed using REDCap® [13, 14] and outcome parameters were calculated using MATBLAB R2020b (The MathWorks Inc., Natick, MA, USA).

### Data collection and processing

Data collection was performed in the Laboratory of Functional Biomechanics at the Department of Orthopaedics and Traumatology at the University Hospital Basel, Basel, Switzerland.

#### Hop performance

Participants completed a pretest for SLH and SH with each leg [29], which consisted of a submaximal forward and sideways hop over 40 cm with hands placed at the hip and controlled single-leg landing with sufficient movement quality and stability [22]. After a successful pretest, participants performed four forward SLHs for maximum distance per leg with unrestricted arm movement [23]. If fewer than two valid hops (single-leg landing, held for at least 2 s) were achieved or if the participants felt that they had not yet reached their maximum distance, up to two additional hops per leg were permitted. The maximum hop distance from the toe of the shoe in the starting position to the heel of the shoe after landing was measured with a tape measure and normalized to body height (% BH).

SHs were performed once per leg with a 3-min rest between legs [23]. The SH consists of a sequence of as many hops as possible over 40 cm within 30 s alternating medially and laterally. The number of valid hops (distance > 40 cm) was recorded.

#### Proprioception

For the active–active (actively predefined and actively reproduced) knee position in the JPS tests [9], participants were seated on a dynamometer (Biodex System 4 Pro, Biodex Medical Systems, Shirley, MA, USA) aligned with the transepicondylar line. Participants were blindfolded and asked to actively move their leg from the starting position (90° knee flexion and 70° hip flexion) to the target knee angle position (60° and 30° knee flexion, respectively), memorize this position, actively reproduce the memorized knee position from the starting position, and press a button on a hand control to confirm the recorded angle (three trials per target angle and leg). The mean of the two smallest absolute differences between the reproduced and predefined knee angle (reproduction error) was calculated.

#### Dynamic postural control

Participants stood with their test leg on the fixed center platform of the YB test kit (Move2Perform, Evansville, IN, USA) with their hands on their hips and pushed the sliding platforms with the free (non-weight-bearing) leg as far as possible in the anterior, posteromedial, and posterolateral directions, holding the maximum position for at least 1 s before returning the free leg to the center and continuing with the next direction (four trials per leg) [37]. If more than two trials were invalid (hands released, heels lifted, free leg pressing on the platform or ground), up to two additional trials were granted. The maximum distance in each direction was recorded and normalized to leg length (LL, distance from anterior iliac spine to medial malleolus), and the composite score [sum of maximum distances in all directions/(3 × LL)] was calculated.

### Data analysis

Statistical analysis was performed in SPSS Version 28.0.1.0 (IBM Corporation, Amonk, NY, USA). All patients who performed a test bilaterally were included in the respective analysis for differences in functional leg performance (comparisons I–II) to obtain uniform testing across both comparisons (within and between groups) with equal group sizes. Normal distribution was visually checked using Q–Q plots. Differences in participant characteristics were tested using parametric or nonparametric analysis of variance (ANOVA). For clinical parameters (only patients, Table 2), nonparametric Mann–Whitney U tests were used.

**Table 2:** Participant information and functional performance in patients after ACL-IB, patients after ACL-R, and controls

Parameter	ACL-IB (*n* = 29)		ACL-R (*n* = 27)		Controls (*n* = 29)		*P*-value
Anthropometrics							
Sex (male/female)	13/16		13/14		13/16		
Age (years)	36.8 (10.6)		37.0 (10.7)		37.0 (10.7)		0.995
Body mass (kg)	73.2 (10.9)		73.1 (14.5)		70.2 (16.6)		0.656
Body height (cm)	172.2 (7.8)		170.5 (7.4)		172.6 (10.8)		0.643
Body mass index (kg/m^2^)*	25.5 [21.2;26.5]*		24.5 [21.9;27.5]*		23.1 [20.4;24.9]*		0.134
Clinical parameters							
Time from injury to surgery (days)*	20 [15;25]*		28 [14;56]*				**0.026**^**f**^
Follow-up (months)*	24.4 [23.6;27.2]*		24.2 [23.6;24.9]*				0.184^f^
Operated leg							
Left	13 (45%)		12 (44%)				
Right	16 (55%)		15 (56%)				
Dominance of involved leg							
Dominant	14 (48%)		11 (41%)				
Non-dominant	15 (52%)		16 (59%)				
Concomitant injuries and surgeries^a^ (e.g., meniscus, MCL, or LCL lesions)	23 (79%)		24 (89%)				
IKDC questionnaire*	85.0 [75.9;92.5]*		82.0 [75.9;87.4]*		97.7 [96.6;100.0]*		** < 0.001**^**d**^; < **0.001**^**e**^; 0.324^f^
TAS at follow-up*	4.0 [4.0;6.0]*		4.0 [4.0;5.0]*		4.0 [3.0;5.0]*		0.329
Functional parameters	Involved	Uninvolved	Involved	Uninvolved	Non-dominant	Dominant	
Hop performance							
SLH distance (% BH) (*N* = 28/26/29)^b^	64.0 (22.7)	68.1 (20.8)	62.8 (15.3)	68.6 (15.8)	68.7 (15.4)	70.0 (14.8)	
SH (*n*)^c^ (*N* = 23/24/29)^b^	19 (11)	22 (13)	20 (14)	22 (12)	21 (12)	22 (13)	
Proprioception							
Joint position sense (*N* = 29/27/29)^b^							
Error 60° flexion (°)	3.0 (2.4)	2.4 (1.7)	2.9 (1.6)	2.8 (1.8)	3.2 (1.8)	2.8 (1.5)	
Error 30° flexion (°)	3.2 (2.3)	2.4 (1.4)	3.3 (1.5)	2.5 (1.7)	3.3 (1.9)	2.9 (2.2)	
Dynamic postural control							
Y Balance (*N* = 29/27/29)^b^							
Anterior (% LL)	63.3 (6.0)	64.6 (5.2)	62.7 (7.4)	63.4 (7.2)	65.8 (6.7)	66.0 (7.5)	
Posteromedial (% LL)	103.6 (11.6)	106.1 (10.2)	102.3 (9.3)	102.9 (8.1)	104.4 (10.0)	104.8 (9.6)	
Posterolateral (% LL)	98.3 (12.3)	98.8 (11.3)	97.1 (8.6)	98.3 (8.9)	96.3 (12.4)	98.3 (12.7)	
Composite score (% LL)	88.4 (9.1)	89.8 (8.3)	87.4 (7.6)	88.2 (6.9)	88.9 (8.5)	89.7 (8.5)	

*ACL-IB* anterior cruciate ligament repair and InternalBrace™ augmentation after proximal ruptures, *ACL-R* anterior cruciate ligament reconstruction, *MCL* medial collateral ligament, *LCL* lateral collateral ligament, *IKDC* International Knee Documentation Committee, *TAS* Tegner activity score, *SLH* single-leg hop, *SH* side hop, *BH* body height, *LL* leg length, *SSD* side-to-side difference (patients: involved–uninvolved; controls: non-dominant–dominant), *CI* confidence interval

Values other than number of participants (*N*) are given as mean (standard deviation) and *P*-values for one-way analysis of variance, if not specified otherwise; distributions are given as number of subjects and percentage of the respective group

*Values are given as median [25;75] percentile and *P*-values for Kruskal–Wallis test with post hoc Mann–Whitney U test, as Q–Q plots revealed no normal distribution in at least one of the groups

^a^Injury- and treatment-related data were obtained from surgical and consultation reports

^b^Number of participants (ACL-IB/ACL-R/controls) who completed a functional test with both legs

^c^Mean values of the number of side hops were rounded down to full jumps

Bold printed results indicate significance (*P* < 0.05)

^d^ ACL-IB versus controls

^e^ ACL-R versus controls

^f^ ACL-IB versus ACL-R

Because a significant difference might not always be clinically relevant or a true difference (and vice versa), we evaluated differences in functional leg performance (I) within-participants’ legs (side-to-side difference, SSD) and (II) between the involved (patients) and the non-dominant (controls) leg according to defined statistical, clinical, and methodological thresholds described below. For each comparison (I–II) and each functional performance test, reaching a statistical, clinical, or methodological threshold was defined as a positive result, respectively. Consequently, no positive results to a maximum of three positive results (exceeding all three thresholds) could be identified in a functional performance test. A strong difference in functional performance was interpreted if three positive results were achieved, a moderate difference if two positive results were achieved, a small difference if one positive result was achieved, and no difference in functional performance if no positive results were achieved in a test.

*Statistical threshold*: statistical significance for comparisons I and II:

(I) Significant leg difference determined by 95% confidence intervals (CI) of SSDs (SSD_patients_ = involved leg–uninvolved leg; SSD_controls_ = non-dominant leg–dominant leg) excluding zero (positive result).
(II) Significant one-way ANOVA between the involved leg of patients and the non-dominant of controls with Bonferroni post hoc tests (*P* < 0.05, positive result).

*Clinical threshold*: values considered as clinically relevant for comparisons I and II:

(I) A leg symmetry of ≥ 90% is considered as normal [1], and in patients, a performance of the involved leg of at least 90% of the uninvolved leg is recommended to return to sports [12]. We calculated a limb symmetry index (LSI = (1 + (SSD/higher leg value)) × 100, where values below/above 100% indicate a deficit/supremacy of the involved (patients) or non-dominant (controls) leg. Because higher values in the JPS test (reproduction error) indicate a lower performance, SSDs in the JPS were multiplied by −1 when calculating the LSI to ensure the above definitions for lower/higher LSI values also in JPS test. According to the literature, an LSI below 90% was used as clinical threshold and considered as a positive result.
(II) Leg differences ≤ 10% within a person were clinically considered as physiological [3, 25]. We calculated the difference between the involved legs of patients and the non-dominant leg of controls (ACL-IB–controls, ACL-R–controls, ACL-IB–ACL-R), and between the involved legs of ACL-IB and ACL-R (ACL-IB–ACL-R) as percentage of the average performance of the non-dominant leg of controls (leg difference/average performance of the non-dominant leg of controls). A negative/positive percentage represents a leg difference with deficit/supremacy of the involved leg of patients compared with controls (patients versus controls) or in ACL-IB compared with ACL-R (ACL-IB versus ACL-R), respectively. Similarly, in this calculation, a leg difference in JPS test was multiplied by −1 to ensure an equivalent interpretation across all functional performance tests. An absolute leg difference greater than 10% of the average performance of non-dominant leg of control subjects indicates a greater leg difference than normally occurs in healthy subjects and was used as a clinical threshold for a positive result.

*Methodological threshold*: absolute differences for comparisons I and II:

- (I–II) Differences greater than the smallest detectable change (SDC) of a test are considered true differences [6, 26]. We compared the absolute leg differences within and between groups with SDC retrieved form the literature. For the normalized parameters in this study (SLH: % BH, YB: % LL), the SDC was divided by the overall groups mean, resulting in the following cut-off values for the respective tests: SLH: > 13.9 cm [23] ≙ 8.1% BH; SH: > 7 hops [23]; JPS: >1.18° × 1.96 × √2=3.8° [27]; YB: 8.7 cm anterior [44] ≙ 9.5% LL; 10.3 cm posteromedial [44] ≙ 11.3% LL; 11.5 cm posterolateral [44] ≙ 12.6% LL; 24.8 cm composite [44] ≙ 9.1% LL. Differences greater than the respective SDC of a test were considered a positive result.

Because unilateral injury may also affect the function of the uninvolved leg [11, 34], using the healthy contralateral leg as sole reference in patients may lead to overestimation of the performance of the involved leg. We calculated (comparison III) the relative number of patients who achieved leg symmetry (defined as LSI ≥ 90%) and a performance of healthy controls (defined as ≥ 90% of the average performance of the non-dominant leg of controls) in SLH, proprioception, and postural dynamic control with the involved leg (patients) or the non-dominant leg (controls) (coded as 1, yes and 0, no). Distributions were compared between groups using chi-squared tests (*P* < 0.05).

## Results

### Participants

Overall, 29 ACL-IB after proximal ruptures, 27 sex- and age-matched ACL-R with semitendinosus tendons (including 4 patients with additional gracilis tendon autograft), and 29 matched controls were included. Deviating from the published protocol [29], we also recruited five patients treated with ACL-R at two other medical centers to generate groups with an acceptable age matching (max ± 4 years). Patients after ACL-IB and ACL-R underwent surgery between February 2017 and April 2019, and between March 2018 and December 2020. Experienced senior surgeons performed ACL-IB (SM, CE, and GP) and ACL-R (SM and CE) surgeries. In both ACL surgeries, a (high) anterolateral portal was used for routine diagnostic arthroscopy and to establish an anteromedial working portal under visual control. In proximal ACL-IB repair, ACL length and tissue quality were confirmed intraoperatively. Then, a 4.5-mm transfemoral drill hole was placed in the anatomical femoral footprint (inside-out) using the anteromedial portal and a high flexed position of the knee. Subsequently, the ACL stump was grasped proximally with one or two sutures depending on tissue quality, which were passed through the transfemoral drill hole for ACL reinsertion to its femoral footprint. The InternalBrace™ augmentation (Arthrex, Naples, Florida, USA) was attached to a femoral flip button. After placement of a 4.5-mm transtibial drill hole in the tibial ACL footprint, the InternalBrace™ was passed through and placed over the femorally reattached ACL. Femoral fixation was realized using a flip button with which the two repair sutures were firmly knotted. For tibial fixation a button and/or a suture anchor was used. In anatomic single-bundle ACL-R, the ipsilateral semitendinosus tendon was harvested using a commercial tendon stripper and further prepared. In four cases, where the four-folded graft diameter was < 7 mm, the gracilis tendon was additionally harvested. Femoral (anteromedial portal, inside-out) and tibial drill holes with the diameter of the prepared hamstring graft were drilled into the femoral and tibial footprints. The graft was passed through the tibia and fixed femorally with a button with or without an additional interference screw. The graft was then tensioned and the position checked for no signs of femoral notching. Finally, a button and/or an interference screw was used for tibial fixation.

Anthropometric parameters did not differ between groups (Table 2). IKDC scores were significantly lower in ACL-IB and ACL-R 2 years postoperatively than in controls, but knee-related activity levels in TAS scores were comparable between groups with a median of 4 (Table 2). Because primary repair surgery must be performed in the early phase after ACL tear [50], time from ACL injury to surgery was significantly shorter in ACL-IB than in ACL-R (*P* = 0.026) (Table 2).

### Functional performance

Two patients after ACL-IB (6.9%) and ACL-R (7.4%), respectively, could not jump over 40 cm with the involved leg, whereas they could with the uninvolved leg. In addition, four patients after ACL-IB and one patient after ACL-R failed the hop pretest with the involved and the healthy contralateral leg. All patients and controls passed the JPS and YB tests (Table 3). Within and between group differences (comparison I–II) are presented in Table 4. The number and interpretation of exceeded thresholds (positive results) in leg comparison of a functional test are presented in Table 5.

**Table 3:** Reasons for failed hop (pre)tests in patients after ACL-IB and patients after ACL reconstruction

*Subject no.*	*Failed hop pretest or test*					
	*SLH*			*SH*		
	*Inv*	*Uninv*	*Reason*	*Inv*	*Uninv*	*Reason*
*ACL-IB*						
28	X	X	No stable landing (both sides)	X	X	No stable landing (both sides), 40 cm distance not reached (both sides)
34				X*		40 cm distance not reached, no confidence
10				X*		40 cm distance not reached, no stable landing
1				X	X	No stable landing (both sides)
13				X	X	Test terminated by the patient after 20 s with the uninvolved leg due to muscular weakness, not willing to perform test with the affected side afterwards
22				X	X	40 cm distance not reached (both sides)
*ACL-R*						
14	X*		Knee pain; no confidence	X*		Knee pain; no confidence
5				X	X	40 cm distance not reached (both sides), no stable landing (both sides), no confidence
32				X*		40 cm distance not reached (both sides)

Note that all controls were able to complete all hop tests

*ACL-IB* anterior cruciate ligament repair and InternalBrace™ augmentation after proximal ruptures, *ACL-R* anterior cruciate ligament reconstruction, *SLH* single-leg hop, *SH* side hop, *Inv* involved leg, *Uninv* uninvolved leg

*Patients were able to jump with the uninvolved leg, but not with the involved leg

**Table 4:** Within (I) and between (II) group differences in functional performance evaluated according to statistical (A), clinical (B), and methodological (C) thresholds

	(I) SSD within groups			(II) involved leg (patient) vs. non-dominant leg (controls)		
	ACL-IB: involved vs uninvolved^a^	ACL-R: involved vs uninvolved^a^	Controls: non-dominant vs dominant^a^	ACL-IB vs Controls^a^	ACL-R vs Controls^a^	ACL-IB vs ACL-R^a^
(A) Statistical threshold	95% CI of SSD exclude zero			Significant one-way ANOVA with *P* < 0.05		
	CI			*P*-value		
Hop performance*						
SLH distance (%BH)	**[− 7.1;− 1.0]**	**[− 9.2;− 2.4]**	[**− **3.0;0.4]	0.445		
SH (*n*)	**[− 7;− 1]**	[**− **4;0]	[**− **2;1]	0.744		
Proprioception						
Joint position sense						
Error 60° flexion (°)	[**− **0.1;1.3]	[**− **0.6;0.9]	[**− **0.5;1.2]	0.846		
Error 30° flexion (°)	[**− **0.1;1.9]	**[0.0;1.6]**	[-0.5;1.4]	0.980		
Dynamic postural control						
Y-Balance						
Anterior (%LL)	[**− **3.1;0.4]	[**− **2.6;1.3]	[**− **1.6;1.3]	0.184		
Posteromedial (%LL)	**[− 4.4;-0.5]**	[**− **2.6;1.2]	[**− **2.0;1.3]	0.735		
Posterolateral (%LL)	[**− **2.5;1.5]	[**− **3.0;0.7]	[**− **4.2;0.2]	0.804		
Composite score (%LL)	**[− 2.8;-0.1]**	[**− **2.2;0.5]	[**− **2.1;0.5]	0.796		

(B) Clinical threshold	LSI < 90%			Absolute leg difference/average performance of non-dominant leg of controls > 10%		
	Mean (SD) or median [25, 75] percentile			Mean (SD)		
Hop performance*						
SLH distance (%)	93.2 (12.8)	91.7 (12.5)	98.0 (6.7)	−6.8 (33.0)	−8.5 (22.2)	1.7 (33.0)
SH (%)	**81.5 (21.0)**	**83.3 (29.1)**	97.3 (22.6)	**−12.6 (52.2)**	−7.3 (66.5)	−5.3 (52.2)
Proprioception						
Joint position sense (%)						
Error 60° flexion (%)	**68.6 [4.4;153.5]**	**83.7 [3.0;142.1]**	112.8 [−4.6;151.8]	6.8 (75.5)	9.1 (49.8)	−2.3 (75.5)
Error 30° flexion (%)	**62.8 [−51.2;130.8]**	**80.0 [−22.3;126.3]**	**55.4 [−58.1;185.7]**	2.9 (67.3)	2.1 (46.1)	0.8 (67.3)
Dynamic postural control						
Y Balance						
Anterior (%)	97.9 (7.0)	99.0 (6.9)	100.0 (5.6)	−3.8 (9.2)	−4.7 (11.2)	0.9 (9.1)
Posteromedial (%)	97.7 (4.7)	99.3 (4.5)	99.7 (4.4)	−0.8 (11.1)	−2.1 (8.9)	1.3 (11.1)
Posterolateral (%)	99.4 (4.9)	98.9 (4.6)	98.1 (5.5)	2.0 (12.7)	0.8 (8.9)	1.2 (12.7)
C omposite score (%)	98.3 (3.9)	99.0 (3.7)	99.1 (3.6)	−0.5 (10.2)	−1.7 (8.6)	1.2 (10.2)

(C) Methodological threshold	Absolute within-leg difference > SDC^*b*^			Absolute leg difference > SDC^*b*^		
	Mean (SD)			Mean (SD)		
Hop performance*						
SLH distance (% BH)	−4.1 (7.7)	−5.8 (8.2)	−1.3 (4.5)	−4.7 (22.7)	−5.9 (15.3)	1.2 (22.7)
SH (*n*)	−4 (6)	−2 (6)	−0 (5)	−2 (11)	−1 (14)	−1 (11)
Proprioception						
Joint position sense						
Error 60° flexion (°)	0.6 (1.7)	0.1 (1.9)	0.3 (2.1)	−0.2 (2.4)	−0.3 (1.6)	0.1 (2.4)
Error 30° flexion (°)	0.9 (2.6)	0.8 (2.0)	0.4 (2.5)	−0.1 (2.3)	−0.1 (1.5)	0.0 (2.3)
Dynamic postural control						
Y Balance						
Anterior (%LL)	−1.3 (4.5)	−0.7 (4.9)	−0.2 (3.8)	−2.5 (6.0)	−3.1 (7.4)	0.6 (6.0)
Posteromedial (% LL)	−2.4 (5.1)	−0.7 (4.8)	−0.3 (4.3)	−0.8 (11.6)	−2.2 (9.3)	1.4 (11.6)
Posterolateral (% LL)	−0.5 (5.2)	−1.2 (4.6)	−2.0 (5.7)	2.0 (12.3)	0.8 (8.6)	1.1 (12.3)
Composite score (% LL)	−1.4 (3.6)	−0.9 (3.2)	−0.8 (3.4)	−0.5 (9.1)	−1.5 (7.6)	1.0 (9.1)

*ACL-IB* anterior cruciate ligament repair and InternalBrace™ augmentation after proximal ruptures, *ACL-R* anterior cruciate ligament reconstruction, *SD* standard deviation, *BH* body height, *LL* leg length, *SSD* side-to-side difference (patients: involved–uninvolved; controls: non-dominant–dominant); *ANOVA* analysis of variance, *CI* confidence interval, *SLH* single-leg hop, *SH* side hop, *SDC* smallest detectable change, *LSI* limb symmetry index

Bold marked values indicate that a difference exceeded a predefined threshold in the respective category: statistical, clinical, methodological (see data analysis section)

*Only patients who passed hop pretests and completed hop tests with both legs were included in this analysis

^a^Negative values indicate lower performance in A compared with B when “A versus B” is given, except for JPS reproduction error, where lower values/errors indicate higher JPS performance

^b^SDC for the respective measurements retrieved from the literature (see data analysis section): SLH, 13.9 cm ≙ 8.1% BH; SH, 7 hops; JPS, 3.8°; Y Balance, 8.7 cm ≙ 9.5% LL anterior, 10.3 cm ≙ 11.3% LL posteromedial, 11.5 cm ≙ 12.6% LL posterolateral, 24.8 cm ≙ 9.1% LL composite

**Table 5:** Evaluation of positive results (exceeded statistical, clinical, and methodological thresholds) in functional performance

	*(I) SSD within groups*			*(II) Involved leg (patient) versus non-dominant leg (controls)*		
	*ACL-IB: involved versus uninvolved*	*ACL-R: involved versus uninvolved*	*Controls: non-dominant versus dominant*^a^	*ACL-IB versus controls*	*ACL-R versus controls*	*ACL-IB versus ACL-R*
Hop performance*						
SLH distance	Small	Small	No	No	No	No
SH	Moderate	Small	No	Small	No	No
*Proprioception*						
Joint position sense						
Error 60° flexion	Small	Small	No	No	No	No
Error 30° flexion	Small	Moderate	Small	No	No	No
*Dynamic postural control*						
Y Balance						
Anterior	No	No	No	No	No	No
Posteromedial	Small	No	No	No	No	No
Posterolateral	No	No	No	No	No	No
Composite score	Small	No	No	No	No	No

*ACL-IB* anterior cruciate ligament repair and InternalBrace™ augmentation after proximal ruptures, *ACL-R* anterior cruciate ligament reconstruction

*Only patients who passed hop pretests and completed hop tests with both legs were included in this analysis

No, small, moderate, or strong differences indicate that 0, 1, 2, or all 3 thresholds were exceeded in the respective parameter

#### Leg differences within participants (I)

No-to-moderate differences (0 to 2 exceeded thresholds) in leg performance were found for ACL-IB and ACL-R, whereas controls had no-to-small differences (0 to 1 exceeded thresholds; Table 5). ACL-IB and ACL-R, on average, jumped significantly shorter (−4.1% BH and −5.8% BH, respectively) with the involved leg than with the uninvolved leg. ACL-IB also completed significantly fewer SH (four hops) with the involved than with the uninvolved leg (Table 4I–A). For SH, both patient groups had an LSI < 90% (Table 4I–B), but their absolute SSDs were lower than the reported SDCs (Table 4I–C). In JPS at 30° flexion, ACL-R had significantly higher knee angle reproduction errors in the involved than in the uninvolved leg (0.8°, Table 4I–A/C). In JPS, almost all LSIs of the reproduction error of all groups were < 90% (Table 4I–B), but the absolute SSDs were smaller than the reported SDC (Table 4I–C). For YB posteromedial and composite scores, ACL-IB performed significantly worse with the involved than with the uninvolved leg (posteromedial: −2.4% LL; composite: −1.4% LL, Table 4I–A). The LSIs of these scores were well above 90% (Table 4I–B) and their absolute values were below the reported SDC (Table 4I–C).

#### Differences between the involved (patients) and non-dominant (controls) legs (II)

A small difference from controls in functional performance was observed in ACL-IB (one exceeded threshold; Table 5). There was no difference between ACL-R and controls or between patient groups. Functional performance was not significantly different between the involved leg of patients and the non-dominant leg of controls (Table 4II–A). The absolute difference between the involved leg of ACL-IB compared with the non-dominant leg of controls was greater than 10% of the average performance of controls in SH (12.6% lower in ACL-IB, Table 4II–B), but smaller than the respective SDC (Table 4II–C).

#### Participants reaching normal leg symmetry and performance (III)

Compared with controls, significantly fewer patients (ACL-IB and ACL-R) achieved an LSI ≥ 90% and a performance in the involved leg of ≥ 90% of the average performance of controls (non-dominant leg) in SLH (*P* < 0.001; Table 6).

**Table 6:** Number (percentage) of patients after ACL-IB, patients after ACL-R, and control subjects reaching an LSI ≥ 90% and ≥ 90% of the average performance of the non-dominant leg of controls^a^With the involved (patients) or non-dominant (controls) leg

Parameter	ACL-IB	ACL-R	Controls	*P*-value*			
				All groups	ACL-IB versus controls	ACL-R versus controls	ACL-IB versus ACL-R
*Single-leg hops*							
SLH distance	11/29 (38%)	8/27 (30%)	22/29 (76%)	** < 0.001**	**0.004**	**0.001**	0.5804
SH	6/29 (21%)	9/27 (33%)	13/29 (45%)	0.148			
*Proprioception*							
Joint position sense							
Error 60° flexion	14/29 (48%)	12/27 (44%)	15/29 (52%)	0.862			
Error 30° flexion	15/29 (52%)	13/27 (48%)	11/29 (38%)	0.551			
*Dynamic postural control*							
Y Balance test							
Anterior	18/29 (62%)	15/27 (56%)	24/29 (83%)	0.075			
Posteromedial	24/29 (83%)	23/27 (85%)	25/29 (86%)	0.932			
Posterolateral	24/29 (83%)	23/27 (85%)	23/29 (76%)	0.845			
Composite score	23/29 (79%)	23/27 (85%)	24/29 (83%)	0.845			

Number of participants reaching leg symmetry and additionally normal leg performance as percentage of the respective overall group size

*ACL-IB* anterior cruciate ligament repair and InternalBrace™ augmentation after proximal ruptures, *ACL-R* anterior cruciate ligament reconstruction, *BH* body height, *LL* leg length, *SLH* single-leg hop, *SH* side hop

*Chi-squared test between distribution of all three groups or the respective two groups as post hoc test (P < 0.05)

^a^Thresholds for 90% of the average performance of controls: SLH, ≥ 61.8% BH; SH, ≥ 19 hops; JPS 60° ≤ 3.5°, 30° ≤ 3.6°; Y Balance, ≥ 59.2% LL anterior, ≥ 94.0% LL posteromedial; ≥ 86.7% LL posterolateral, ≥ 80.0% LL composite score

## Discussion

We aimed to determine differences in functional leg performance in hops, knee proprioception, and dynamic postural control within and between patients 2 years after InternalBrace™-augmented ACL repair, patients 2 years after ACL-R, and controls using statistical, clinical, and methodological thresholds, as well as to compare the number of participants achieving normal leg symmetry and normal leg performance between groups. According to the number of exceeded predefined thresholds (see method section), we observed no-to-moderate leg differences within ACL-IB and within ACL-R. We noted no-to-small differences within the legs of controls, no leg differences between ACL-IB and ACL-R (involved legs), no-to-small leg differences between ACL-IB and controls, and no leg differences between ACL-R and controls in functional performance including hops, knee proprioception, and dynamic postural control 2 years after surgery. Moreover, in each patient group, two patients were able to hop 40 cm only with their uninvolved leg but not with their involved leg, and in SLH, fewer patients (ACL-IB and ACL-R) than controls reached leg symmetry and leg performance of the non-dominant leg of healthy controls.

### Differences within and between ACL-IB, ACL-R, and healthy controls

#### Hop performance

In agreement with our study, Leister et al. [25] reported no clinically relevant difference in the LSI of ACL-IB in SLH, but in contrast to our study, a clinically relevant difference in LSI of matched ACL-R (hamstring autografts) in SLH 13 months postoperatively. Also similarly to our results, these authors observed a clinically relevant difference in LSI of less than 90% in ACL-IB and ACL-R in SH [25]. Values in this range have been associated with lower patient-reported knee function and degenerative changes [33] or were used as no return to sports criteria [12]. Nevertheless, no significant or relevant differences were found between the matched patient groups in either this study or ours. Moreover, a comparable amount of ACL-IB and ACL-R (6.9% versus 7.4%) passed the hop pretests (jumping laterally over 40 cm with one leg) with their uninvolved leg only, but not with their involved leg. This corresponds to an actual deficit of 100% in the involved leg compared with the uninvolved leg and controls (all controls completed all pretests). While Leister et al. [25] did not report such high deficits in the involved leg for patients after ACL-IB or ACL-R, other authors reported such high deficits for 4 of 81 (4.9%) patients 1 year after ACL-R (hamstring autografts) in SH [33]. Unfortunately, the reasons for not jumping were not described [33]. Our finding that some patients were unable to jump with at least one leg could be explained by the lower activity level (median, TAS 4) compared with the other study investigating hop performance in ACL-IB and matched ACL-R (median, TAS 6.0 [25]).

Compared with controls, only ACL-IB achieved a clinically relevant but statistically nonsignificant threshold in SH, whereas patients after ACL-R just missed these thresholds, with only one more hop. These differences could be a consequence of the possibly underpowered ACL-R study group compared with ACL-IB or control group. Furthermore, if our findings on normal leg symmetry and performance are taken into account, it appears that both groups of patients (also ACL-R) have deficits not only in the involved leg compared with the uninvolved leg, but also with the matched control legs. Consistent with this assumption, significantly lower leg symmetry in LSI in SLH has also been reported for both ACL groups compared with LSI of controls in the literature [25].

In agreement with the literature, we consider the observed failures in hop pretests, small-to-moderate leg differences within ACL-IB, small differences within ACL-R, and small differences compared with controls as relevant deficits with functionally compromised hop performance 2 years after ACL surgery.

#### Proprioception

We observed small differences within ACL-IB, small-to-moderate differences within ACL-R, and no-to-small differences in controls in the JPS test. Higher reproduction errors have been reported in the literature for active and passive JPS after ACL-R (with different types of autografts) compared with controls, indicating impaired proprioceptive performance up to 2 years postoperatively [8]. However, the results in active–active JPS 9–24 months after ACL-R with only hamstring tendon autografts are controversial [9, 42, 47]. The differences between the studies that found a significant difference between ACL-R and controls [42, 47] and our study results without significance after ACL-R compared with controls may be explained by methodological differences or different activity levels of patients [39, 46]. Since JPS reproduction error describes very small values with a very high relative variability, and only 38–52% of controls achieved an LSI ≥ 90% and a leg performance ≥ 90% of their average performance in the JPS, we consider the use of the LSI, including a 90% cut-off, as clinically not useful. Clinically relevant thresholds for the absolute achieved reproduction error of a leg (< 5°) have been described in the literature [38]. The average of all legs in our study was well below this value, including both legs of our patients after ACL-R, despite significant differences in JPS error at 30° knee flexion. Moreover, the SSDs were comparable to those reported in a review paper [10]. These authors and others [38] questioned whether the reproduction error or its SSDs in the JPS are relevant to knee function and performance. Therefore, we do not consider the observed leg differences in patients and controls to be relevant. These results suggest that there are no relevant differences in proprioceptive function after ACL-IB and ACL-R, and they neither support nor refute the suggestion that proprioceptive function is preserved after ACL-IB [50], nor that proprioception is impaired after ACL-R using hamstring tendon autografts. To the best of our knowledge, the (negative or positive) influence of the synthetic InternalBrace™ on the mechanosensory receptors in the ACL or on the transmission of stimuli has not been investigated and may be an area for further research.

Nevertheless, despite the lack of differences in assessment of methodological thresholds across all comparisons and without relevant differences in JPS, we must consider that the JPS test alone may not be sensitive enough to detect proprioceptive deficits. Proprioception is presumably also facilitated by proprioceptors in other joint tissues or surrounding muscles, and the JPS reflects only one aspect of proprioception [40]. Moreover, weight-bearing assessment of proprioception may be more functional [46] and could explain the possible decreased performance in mediolateral hops despite good proprioceptive results. Hence, further investigation of proprioception in ACL-IB compared with ACL-R and controls is warranted.

#### Dynamic postural control

The similar YB performance in both legs after ACL-R is consistent with recent literature 2 to 3 years after ACL-R surgery [31]. Although statistically significant, SSD in YB posteromedial and composite scores after ACL-IB were well above clinical and below methodological thresholds. In addition, conflicting results regarding an association between asymmetry in the YB posteromedial score of ≥ 4 cm and asymmetry in the composite score of ≥ 12 cm with future injuries were reported in one review [36]. The absolute asymmetry in our ACL-IB group was lower than these values (for comparison, absolute SSD: posteromedial: 2.1 cm; composite: 3.8 cm). Therefore, we consider the observed small leg differences within ACL-IB to be irrelevant. In contrast to our results, other authors reported lower anterior YB score in the involved leg compared with controls after ACL-R (patellar or hamstring tendons) [31]. This was explained by the inability to resist anterior tibial translation, possibly due to higher quadriceps activity in this direction [21] or the inability to compensate for this translation by presumably weakened hamstring muscles after ACL-R with hamstring tendon autografts [35]. Although we could not confirm these results, the largest discrepancy between both ACL patient groups and control subjects in this YB direction in comparison of number of participants achieving normal leg symmetry and performance may indicate difficulties for patients. To achieve comparable anterior distance as the control subjects, our patients may have used different movement strategies, as reported in patients 2.6 years after ACL-R when performing the anterior reach distance test of the modified Star Excursion Balance test [5]. Hence, examining not only performance, but also kinematics during such a complex task may provide better insight into potentially different mechanisms or strategies between ACL-IB and ACL-R.

#### Normal leg symmetry and performance

Across all tasks, the lowest percentage of patients with leg symmetry and normal leg performance were observed in hops, with lower percentage in SH than SLH in ACL-IB and controls. This has also been reported in the literature when only leg symmetry was studied in patients 1 year after ACL-R [33]. In that study, only 36% of patients achieved 90% of the performance of the contralateral leg in SH, whereas 62% achieved this level in SLH [33]. These findings reflect the higher complexity of lateral jumps, which may be supportive or even more informative for identifying deficits after ACL surgery. The finding in our study that in SLH fewer patients than controls reached leg symmetry and fewer patients achieved leg performance of the non-dominant leg in controls suggests incomplete rehabilitation in hops 2 years after ACL surgery, regardless of ACL-IB or ACL-R.

Overall, none of the differences examined were below the respective SDC of any test, which calls into question the differences we considered relevant based on reliability and detectability. Further studies defining clear criteria for relevance and methodological verifiability in the specific ACL-R and ACL-IB populations are needed. In addition, complementary analyses of movement execution (representing movement quality) would be useful to provide further information not only on the extent of performance (quantity), but on how performance was achieved.

### Strengths and limitations

The main strengths of our study were that all groups were matched for age and sex and did not differ in their knee-specific activity level (TAS). Furthermore, the number and type of concomitant injuries and surgeries in ACL-IB and ACL-R were comparable between groups, as reported in another manuscript related to the umbrella project [30]. Consequently, differences between patients can mainly be attributed to the surgery and not to concomitant injuries or surgeries. For the first time, we provided data on functional leg performance (in terms of proprioception and dynamic postural control) after ACL-IB, and assessed the performance outcomes using statistical, clinical, and methodological thresholds. A limitation of our study is that we excluded patients from the hop test analysis if they failed the pretest to obtain consistent data for our analysis within and between groups. This may have resulted in an overestimation of patients’ hop performances and masked the presence of positive results on the other defined hop parameters thresholds. Moreover, the SDCs were derived from the literature (e.g., healthy population) and hence may not be representative for the patient populations studied. The exclusive inclusion of patients with proximal ACL tears in ACL-IB and all types of ruptures in ACL-R may have biased our results. Because of the moderate knee-related activity level of our participants and the inclusion criteria for patients, the results may not be generalizable to highly active or professional athletes or patients after ACL reconstruction with other tendon grafts. Finally, although patients completed standard physical therapy, the duration and adherence to therapy were not recorded, which may have influenced our results and the activity level of participants.

### Clinical implications

Our results show no differences (assumed to be relevant) in functional leg performance in hops, proprioception, and dynamic postural stability between ACL-IB for proximal ruptures and ACL-R with hamstring tendon autografts. The comparable functional performance outcome, but less invasive ACL repair surgery, highlights the potential of augmented ACL repair next to ACL-R for a specific subgroup of patients represented in our study (e.g., proximal ruptures, adults with moderate knee-specific activity level, for example, TAS of 4). The presumed advantage of augmented ACL repair providing comparable functional performance with preserved knee structures (i.e., native ligament and muscle–tendon complex) must be weighed against the reported higher rerupture rates compared with the gold standard ACL-R [7]. However, with careful patient selection (i.e., patients aged ≥ 21 years [7], TAS score ≥ 7 [15], corresponding to recreational activities such as soccer, rugby, ice hockey, and basketball, or competitive activities such as skiing and gymnastics) it might be possible to reduce the rerupture rates. Apart from this, in our study, neither ACL-IB nor ACL-R achieved the full functional performance level of healthy controls 2 years postoperatively, questioning whether ACL-IB or ACL-R surgery can restore full lower leg function after ACL tear.

## Conclusions

We found no-to-moderate differences within patients 2 years after augmented ACL repair for proximal ruptures and after ACL-R, and no differences between patient groups in functional leg performance of hop, knee proprioception, and dynamic postural control using statistical, clinical, and methodological thresholds. However, the differences and performances of the patients compared with age- and sex-matched controls with comparable activity level show a clinically relevant leg asymmetry and a persistently impaired functional performance in hops in both ACL groups. Further investigations on functional outcome after ACL-IB in direct comparison to ACL-R, including joint mechanics and associated risks, are warranted.

## Data Availability

All data generated or analyzed during this study are included in this published article. Source data are available by reasonable request to the corresponding author.

## Abbreviations

ACL: Anterior cruciate ligament
ANOVA: Analysis of variance
CI: Confidence interval
IB: InternalBrace™ (Arthrex Inc.)
IKDC: International knee documentation committee
JPS: Joint position sense
LSI: Limb symmetry index
R: Reconstruction
SDC: Smallest detectable change
SSD: Side-to-side difference
TAS: Tegner activity score
